# Caffeine Prevents Transcription Inhibition and P-TEFb/7SK Dissociation Following UV-Induced DNA Damage

**DOI:** 10.1371/journal.pone.0011245

**Published:** 2010-06-21

**Authors:** Giuliana Napolitano, Stefano Amente, Virginia Castiglia, Barbara Gargano, Vera Ruda, Xavier Darzacq, Olivier Bensaude, Barbara Majello, Luigi Lania

**Affiliations:** 1 Department of Structural and Functional Biology, University of Naples Federico II, Naples, Italy; 2 Naples Oncogenomic Center (NOGEC), Naples, Italy; 3 Institut de Biologie de l'Ecole Normale Supérieure (IBENS), CNRS UMR 8197, Paris, France; University Medical Center Hamburg-Eppendorf, Germany

## Abstract

**Background:**

The mechanisms by which DNA damage triggers suppression of transcription of a large number of genes are poorly understood. DNA damage rapidly induces a release of the positive transcription elongation factor b (P-TEFb) from the large inactive multisubunit 7SK snRNP complex. P-TEFb is required for transcription of most class II genes through stimulation of RNA polymerase II elongation and cotranscriptional pre-mRNA processing.

**Methodology/Principal Findings:**

We show here that caffeine prevents UV-induced dissociation of P-TEFb as well as transcription inhibition. The caffeine-effect does not involve PI3-kinase-related protein kinases, because inhibition of phosphatidylinositol 3-kinase family members (ATM, ATR and DNA-PK) neither prevents P-TEFb dissociation nor transcription inhibition. Finally, caffeine prevention of transcription inhibition is independent from DNA damage.

**Conclusion/Significance:**

Pharmacological prevention of P-TEFb/7SK snRNP dissociation and transcription inhibition following UV-induced DNA damage is correlated.

## Introduction

In mammalian cells DNA-damage response (DDR) induces a variety of cellular processes, including DNA repair, cell cycle arrest and apoptosis. At transcriptional level the DDR induces a global reprogramming of gene expression with the vast majority of genes being repressed [1]–[3].

The DDR-mediated mechanisms underlying the transcriptional reprogramming following DNA damage are largely unknown. A common feature of the cellular response to DNA-damaging radiation such as X-ray, gamma rays, ultraviolet irradiation (UV) and drug-induced DNA damage is the induction of a high phosphorylation degree of the largest subunit (Rpb1) of Pol II and proteasome-dependent degradation of Rpb1 [4]–[6]. The carboxyl-terminal-domain (CTD) of Rpb1 serves as a scaffold for the interaction of a wide range of factors that orchestrate transcription and co-transcriptional processes [6]. Recruitment of transcription and processing factors is closely linked to CTD phosphorylation at Ser-5 and Ser-2 positions, which is concurrent with transition of the Pol II complex from initial promoter clearance to productive elongation. Thus, it is not of a surprise that changes in the phosphorylation state of Rbp1-CTD have a striking effect on gene expression. Several kinases contribute to CTD phosphorylation, in particular, Ser-2 phosphorylation is mediated by CDK9, which is required for the Pol II complex to enter the mode of productive elongation. CDK9 is the catalytic subunit of the positive transcription elongation factor b (P-TEFb), which consists of a complex between CDK9 and its cyclin T partner. Moreover, P-TEFb integrates mRNA synthesis with histone modification, pre-mRNA processing, and mRNA export [7]–[11]. P-TEFb is found within a cell in two forms referred to as large (LC) and free small (SC) complexes. The kinase active SC complex contains CDK9 and cyclin T1, which is the predominantly associated cyclin. In the LC inactive complex P-TEFb is associated with the 7SK snRNP that contains HEXIM1 or 2 and the 7SK RNA-interacting BCDIN3 and LARP7 proteins [12]–[16]. The dynamic partitioning of P-TEFb between the complexes constitutes a functional equilibrium that can be perturbed by transcription arrest, and hypertrophic signals, and loss of LARP7 function shifts the P-TEFb equilibrium toward the active state and causes P-TEFb-dependent malignant epithelial transformation [17]. Most importantly a variety of DNA-damaging agents induce a rapid release of the P-TEFb LC complex with a concomitant accumulation of the active P-TEFb complex.

In this study we found that caffeine prevents disruption of P-TEFb LC, and transcription following DNA damage induced by UV.

## Results

### Caffeine prevents dissociation of the large inactive P-TEFb complex following UV irradiation

Initiation of DDR by agents such as UV, Camptothecin, Actinomycin D causes a rapid release of P-TEFb from the LC, which is concurrent with Rbp1-CTD hyper-phosphorylation and transcription repression [12], [13], [18].

**Figure 1:**
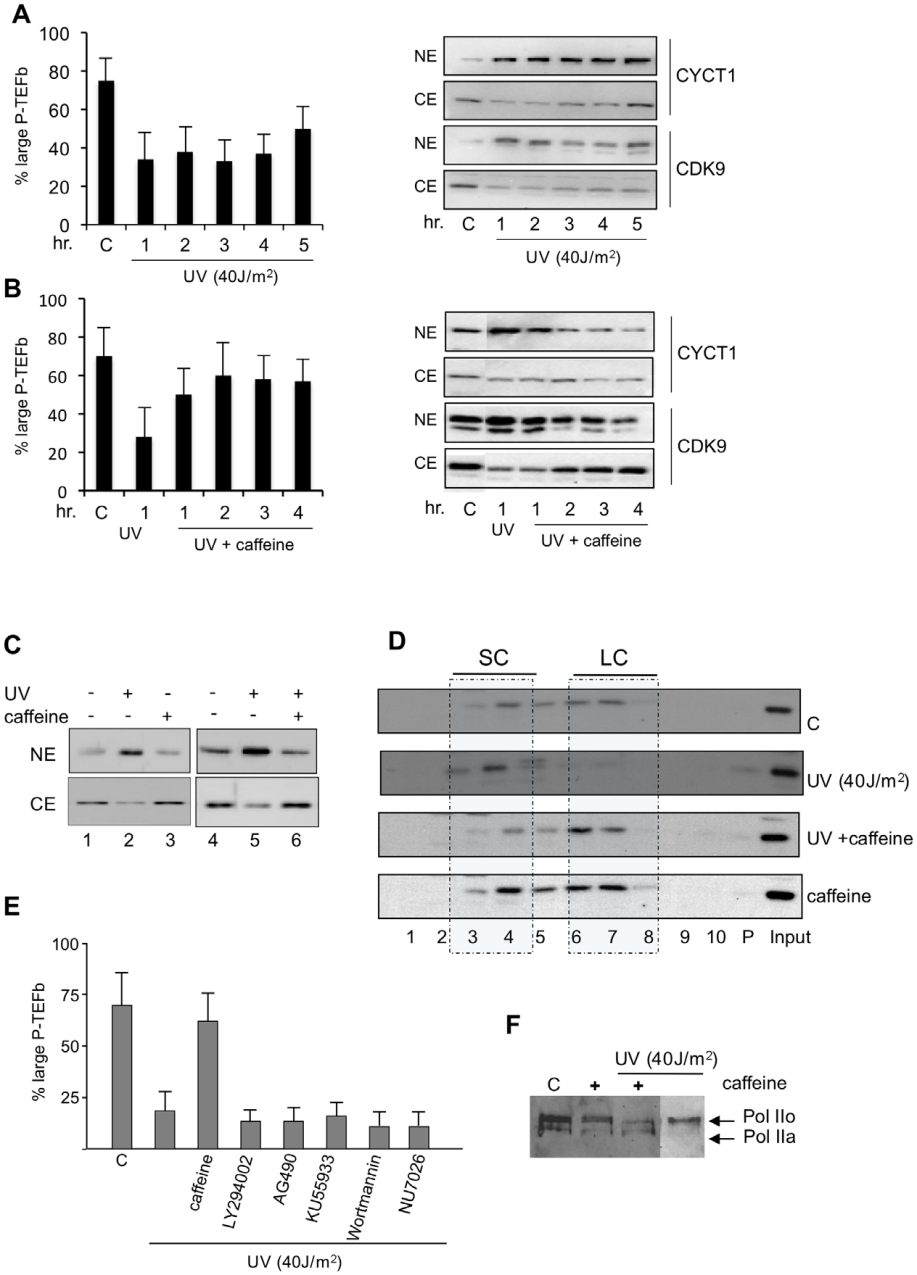
Caffeine prevents dissociation of the large inactive P-TEFb complex following UV irradiation. A) HeLa cells were irradiated with UV (40J/m^2^) and at the indicate times (hours) after irradiation cellular proteins were extracted with different buffers as described in the text, and immunoblotting was performed on low cytosolic extracts (CE) and high-salt nuclear extracts (NE) to detect the percentage of CDK9 and CYCT1 in the free and large form of P-TEFb complex as indicated. On the left a graph reports the relative quantification of the immunoblots as percentage of large P-TEFb complex, graphs are representative of at least four independent experiments, error bars represent standard deviation from the mean (n = 3–4). The percent of P-TEFb in large complex (low salt or CE) was calculated as a fraction of total amount of P-TEFb (both in CE and NE). On the right western blots from a single experiment are shown. B) Carrier dimethyl sulfoxide (control, C), or caffeine (2mM), were added to HeLa cells 120 min prior UV irradiation, cells extracts were prepared after different times (hours) of recovery and processed as in A. The relative quantification of the percentage of large P-TEFb is shown on the left, graphs are representative of at least four independent experiments, error bars represent standard deviation from the mean (n = 3–4). On the right western blots from a single experiment are shown. C) HeLa cells were treated with caffeine for 2 hrs and, as indicated were irradiated with UV (40J/m^2^) and cell extracts were prepared after 1 hr of recovery and immunoblots with anti-CDK9 were performed. D) Whole cell lysates of Hela cells untreated (C) or UV irradiated in the presence or absence of caffeine (2mM, added to cells 120 min prior UV irradiation), or treated with caffeine alone (2mM 2 hrs of treatment), were subjected to glycerol gradient sedimentation (5%–45%), and the fractions analyzed by immunoblotting with CDK9 antibody, the relative position of small complex (SC) and large (LC) are indicated, (P) pellet. Whole cell extract from irradiate cells were prepared after 1 hr. of recovery. E) Carrier dimethyl sulfoxide (control, C), or caffeine (2mM), LY294002 (10µM), AG490 (100µM), KU55933 (20µM), wortmannin (50µM), NU7026 (20µM) were added to HeLa cells 120 min prior UV irradiation, after 1 hr of recovery the percent of P-TEFb in large complex (low salt or CE) was calculated as a fraction of total amount of P-TEFb (both in CE and NE). These experiments were repeated 3–4 times and quantification of data (mean ± SD) is shown. F) UV induces RNAPII hyperphosphoryation which is prevented by caffeine. Western blotting analyses of RNAPII in HeLa cells irradiated with UV (40J/m^2^) in the presence or absence of caffeine (2 hr of pretreatment) with 1 hr of recovery, or treated with caffeine alone were analyzed with RNAPII 8WG16 antibody.

DDR involves activation of the PIKK-family protein kinases, ATM, ATR and DNA-PK; as these kinases impinge on many processes including transcription, we sought to evaluate the putative relationship between P-TEFb LC disruption and DDR activated kinases. To investigate this possibility, we treated cells with the broad PIKK inhibitors caffeine. The drug was added to cells 2 hrs prior to UV treatments and the large and free forms of P-TEFb were analyzed after different times (hrs) of recovery following UV treatment in the presence or absence of caffeine (Fig. 1 A and B). The ratio between P-TEFb SC and LC forms was analyzed in a semi-quantitative manner with a protein extraction protocol based on differential salt extractability of the two complexes [18], [19]. Cells were lysed with the low salt buffer to generate cytosolic extracts CE (containing the large form of P-TEFb) and a nuclear pellet NE (containing the free form of P-TEFb). Both CE and NE extracts were analyzed by western blotting for the presence of CDK9 ad CYCT1. The percent of P-TEFb in large complex (low salt or CE) was calculated as a fraction of total amount of P-TEFb (both in CE and NE). Shortly after UV irradiation (1 hr) P-TEFb LC disruption was found. However, exposure to caffeine effectively prevented UV-mediated dissociation of LC P-TEFb.

P-TEFb dissociation after UV treatment could be detected only after 60′ of recovery, and the protective effect of caffeine was evident after 60′ of recovery (Figure S1) Moreover, caffeine alone did not significantly modulated P-TEFb equilibrium (Figure 1 C and D and Figure S1).

To further validate the protective effect of caffeine, P-TEFb equilibrium was evaluated by glycerol gradient fractionation of cell lysates from samples treated with UV in the presence or absence of caffeine (Fig. 1D). Accordingly with the findings observed with the differential salt extractability protocol, caffeine effectively prevented UV-induced P-TEFb LC dissociation. Moreover, a similar protective effect of caffeine treatment on P-TEFb LC dissociation was seen in U2OS and in p53−/− H1299 cells (data not shown).

Because caffeine is a large spectrum PIKK inhibitor we tested more specific kinase inhibitors such as the specific ATM inhibitor (KU55933), wortmannin, and DNA-PK inhibitor (NU7026). In addition, the Akt inhibitor (LY294002) and the Jak inhibitor (AG490) were also included in our studies because previous works showed that Akt and Jak-dependent pathways might be involved in the control of P-TEFb equilibrium [20], [21]. Of the different inhibitors used only caffeine prevented P-TEFb LC dissociation (Fig. 1E).

Because Rpb1-CTD is a major substrate of P-TEFb activity we look at the phosphorylation status of Rpb1 after UV damage in the presence or absence of caffeine. As shown in Fig. 1, panel F, UV induced hyperphosphorylation of Rbp1, conversely caffeine pretreatment restored, at least in part, the cellular content of Rpb1 isoforms, suggesting that prevention of P-TEFb release from LC reduces the UV-induced hyperphosphorylation of Rbp1-CTD, while caffeine alone did not significantly affect the phopshorylation status of Rbp1-CTD. Collectively these findings demonstrated that caffeine effectively prevents P-TEFb LC dissociation following UV DNA damage.

### Caffeine prevents UV-induced transcriptional repression

**Figure 2:**
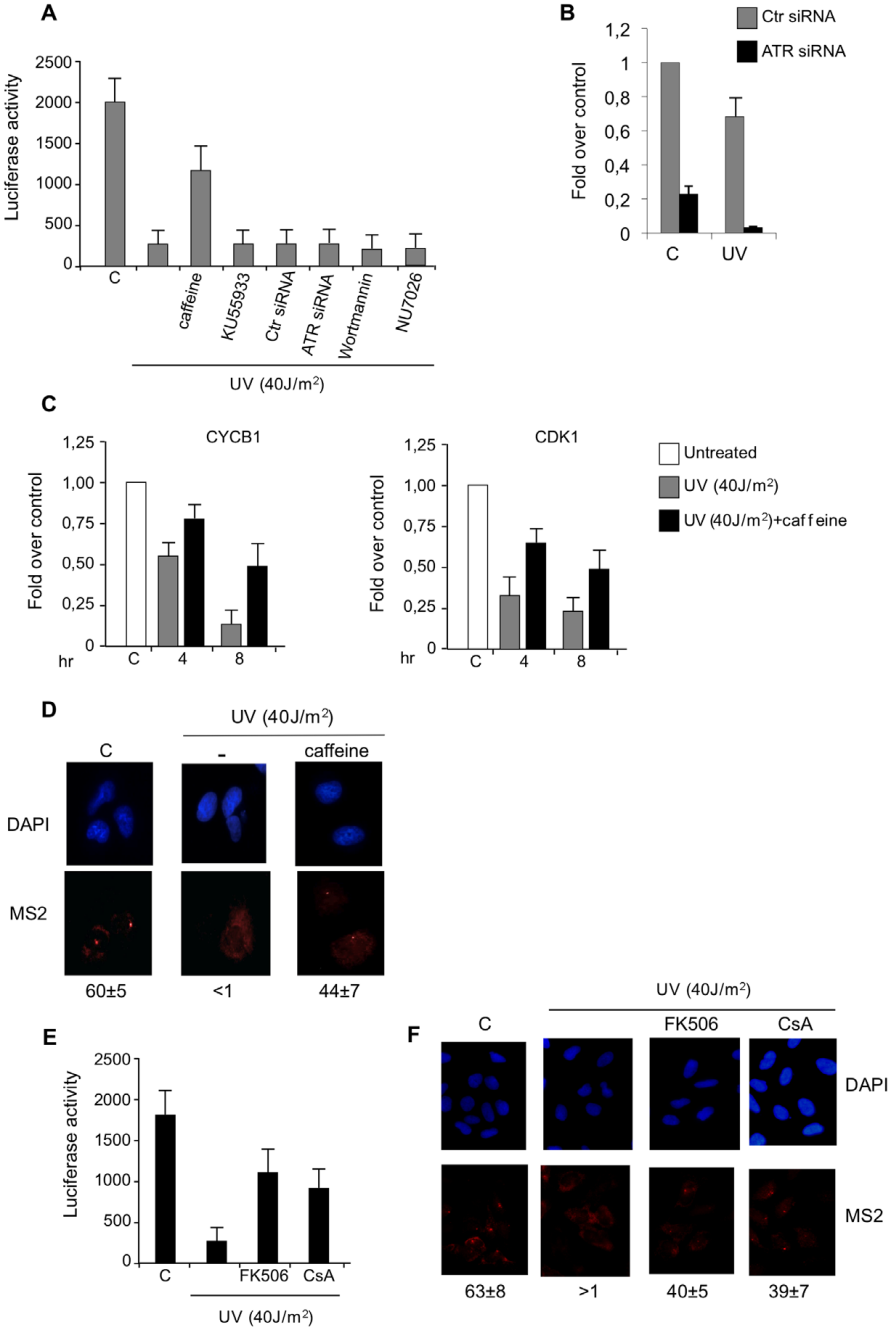
Caffeine prevents UV-induced transcriptional repression. A) 5BCP9F cells were pre-treated with control vehicle (C) or caffeine (2mM) for 120 min. Cells were then irradiated with UV light and subsequently doxycycline was added at the final concentration of 2µg/ml. Luciferase assays were performed after 4 hours. In siRNA experiments ATRsi and Ctrsi RNAs were transfected into 5BCP9F cells 48 hr before treatments. Cells were subjected to UV light and doxycycline was added. Luciferase activities were determined 4 hours after DNA damage. Graphs are representative of at least four independent experiments, error bars represent standard deviation from the mean (n = 3–4). B) 5BCP9F cells were transfected with control (Ctr) or ATR siRNAs for 48h and then treated with UV as indicated; RNA was extracted and subjected to qRT-PCR using primers for ATR mRNA. C) Real-time RT-PCR analysis of gene expression in response to UV irradiation in the presence or absence of caffeine. Cells were harvested at the indicated time after UV treatment, total RNA was isolated, and qRT-PCR was performed with primers specific for the indicated mRNAs. Values were normalized to those of GUS mRNA and are expressed as fold reduction over untreated cells. Results shown are the average of two different experiments performed with triplicate PCRs along with SD (n = 2). D) 5BCP9F cells were treated with doxycycline for 3 hours, caffeine (2mM) was added to the same doxycycline-containing medium and left for additional 2 hours before UV irradiation. After 1 hour of recovery cells were fixed and hybridized with fluorescent (Cy3) MS2 DNA probe and stained with DAPI. The percentage of MS2 positive cells (from 3 independent experiments) is reported along with the standard deviation from the mean. E) 5BCP9F cells were pre-treated with control vehicle (C) or FK506 (3µg/ml) or CsA (10µM) for 120 min as indicated. Cells were then irradiated with UV light and subsequently doxycycline was added at the final concentration of 2µg/ml. Luciferase assays were performed after 4 hours. F) 5BCP9F cells were treated with doxycycline for 3 hours, FK506 (6µg/ml) or CsA (10µM) was added to the same doxycycline-containing medium and left for additional 2 hours before UV irradiation. After 1 hour of recovery cells were fixed and hybridized with fluorescent (Cy3) MS2 DNA probe and stained with DAPI.

As any treatment that results in an arrest in transcription leads to P-TEFb LC dissociation, we sought to determine the functional consequences of caffeine treatment on DNA damage induced transcriptional arrest. To this end we used a U2OS Tet-on cell line (U2OS/pTet-globin-Luc-CFP-24MS2, named 5BCP9F cells) harboring a stable integration at a single locus of approximately 30 repeats of a gene cassette each containing tetracycline-inducible promoter driving the synthesis of a transcript with the beta-globin first exon placed before the Luc cDNA followed by a 24 MS2 repeats in its 3′ untranslated region. Reverse tet transactivator (rtTA), in the presence of doxycycline, drives gene expression from the minimal CMV promoter that can be monitored by Luciferase measurements or by RNA FISH with antisense MS2 probe (Ruda V. et al. in preparation). We found that UV treatment of 5BCP9F cells, repressed Luciferase expression about 10 fold compared to untreated cells (Fig. 2A). Interestingly, pretreatment with caffeine prevented the drop in luciferase activity in UV treated cells, indicating that caffeine-mediated prevention of P-TEFb dissociation correlates with a decreased UV-induced transcriptional repression (Fig. 2A).

Caffeine is a relatively unselective drug and it has been shown to inhibit multiple DDR activation of the multiple PIKK-family protein kinases, such as ATM, ATR and DNA-PK. Further analysis aimed at examining the involvement of ATM and/or ATR pathways showed that a specific ATM inhibitor (KU55933), as well as specific siRNA silencing of ATR failed to prevent the UV-induced decrease in pTet-on-CMV—globin-Luc-24MS2 expression (Fig. 2 panels A, B). Moreover, pretreatment with additional pharmacological inhibitors such as LY294002, NU7026 and Wortmannin did not protect Luciferase activity following DNA damage (Fig. 2A). These data indicate that the DDR activation of the PIKK-family protein kinases is not responsible for the caffeine-mediated protection of transcription.

To further correlate P-TEFb dissociation and transcriptional gene repression we looked at cellular genes that are repressed following DNA-damage such as CDK1 and CYCB1. In agreement with previous reports both CYCB1 and CDK1 were rapidly down-regulated by UV treatment [22], however caffeine pretreatment prevented CDK1 as well as CYCB1 transcription inhibition (Fig 2 C).

Finally, we sought to address the effect of caffeine on transcription at chromosomal level of the single integrated gene cassette of the Luc cassette. To this end RNA-FISH experiments were performed using MS2-labelled anti-RNA probe in cells after UV-damage in the presence or absence of caffeine (Fig. 2 panel D). The RNA-FISH data validated the protective effect of caffeine in UV induced transcription arrest. It is important to note that caffeine effect is not fully sufficient to protect transcription levels comparable to that in untreated cells (Fig. 2), suggesting that caffeine-independent mechanisms contribute to DNA damage transcription arrest.

It has been recently shown that UV light induces release of P-TEFb from the large complex by cooperative action of calcium protein phosphatase 2B (PP2B) protein phosphatase 1 (PP1a), and treatment with PP2B inhibitors FK506 and cyclosporine A (CsA) prevented UV-mediated dissociation of LC P-TEFb [23]. Because caffeine is known to affect Ca^2+^ signaling [24] we sought to determine the effect of FK506 and cyclosporine A (CsA) on transcription following UV exposure. We found that both pharmacological treatments prevented transcription inhibition (Fig. 2 panels E and F). However, unlike caffeine high concentrations of both drugs were required for such effects.

### Caffeine does not attenuate UV-induced γ-H2AX foci

**Figure 3:**
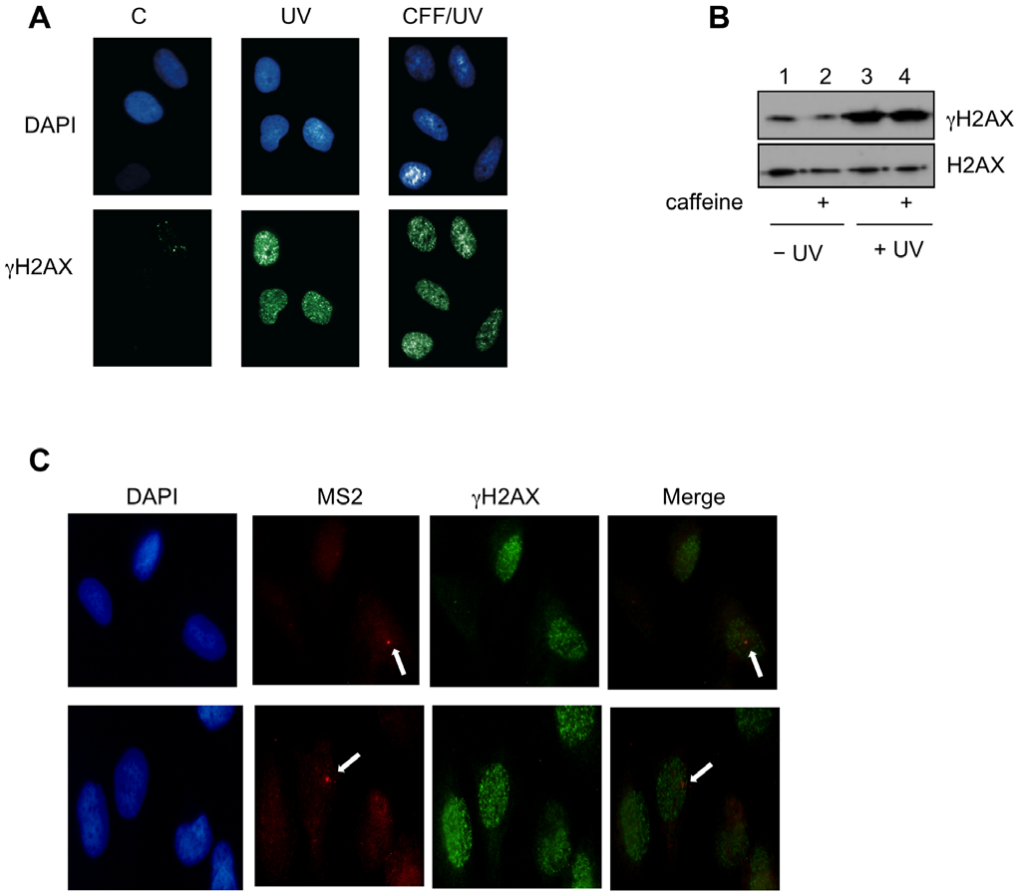
Caffeine prevents transcription inhibition independently from DNA damage. 5BCP9F cells were pre-treated (2hr) with caffeine and exposed to UV 40J/m^2^); after 1 hour of recovery cells were then analyzed by immunofluorescence (A) or immunoblotting (B) with the H2AX and γ-H2AX antibodies, as indicated. C) Doxycycline was added to 5BCP9F cells and left for 2 hours. Caffeine was then added to the same doxycycline containing medium and left for additional 2 hours. Cell were fixed 1 hour after UV irradiation and hybridized with fluorescent (Cy3) MS2 DNA probe (MS2, red) and subjected to immunofluorescence with the γ-H2AX antibody (green) and stained with DAPI. The merge signals (red vs green) from two different experiments are shown.

We sought to determine whether the property of caffeine to prevent P-TEFb LC release and prevents transcription inhibition could be due to a general protective effect of this drug in DNA damaged cells. However, as described above, we found that pharmacological inhibition of DNA damaging signal-regulated kinases do not prevent P-TEFb LC release nor rescue transcription, suggesting that UV-activated kinases such as DNA-PK or ATR/ATM, do not significantly contribute to regulation of equilibrium of the SL and LC P-TEFb complexes (Fig. 1D and 2A). To further elucidate the transcriptional protective function of caffeine in UV irradiated cells, we sought to determine the presence of DNA damage signature in caffeine pretreated cells after UV irradiation. As largely expected induction of γ-H2AX foci, a marker of DNA damage, was detected upon UV irradiation. However, as shown in Fig 3 (A and B) caffeine treatment did not influence the induction of γ-H2AX in UV-treated cells, suggesting that the protective transcriptional effects exerted by caffeine in UV-treated cells occurs in the presence of DNA damage. To further substantiate such hypothesis we performed RNA FISH analysis in caffeine treated cells after UV irradiation. As shown in Figure 3C, we found that caffeine pretreatment prevents transcription inhibition, and most importantly, fluorescence microscopy analysis performed in caffeine treated cells after UV irradiation demonstrated that the RNA FISH signals colocalize with γ-H2AX foci. These results suggest that caffeine prevents transcription inhibition in UV irradiated cells independently from DNA damage.

### Caffeine prevents UV-induced repression of Pol II elongation

**Figure 4:**
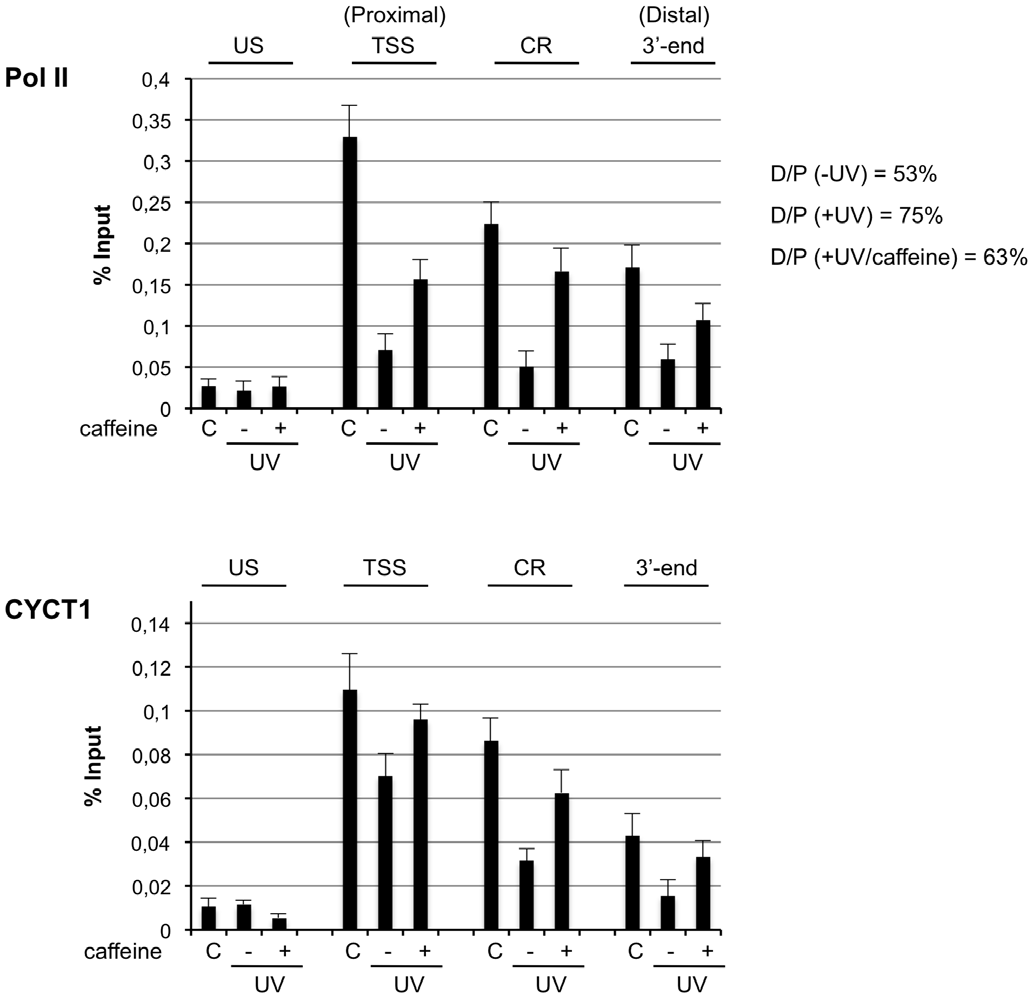
Levels of chromatin bound Pol II and P-TEFb. 5BCP9F cells were treated with doxycycline for 4 hrs to induce expression of Luc gene, then cells were exposed to UV in the presence or absence of caffeine (2 hours pretreatment), after 1 hr of recovery following DNA damage chromatin was prepared and subjected to chromatin immunoprecipitation. Levels of total RNAPII were analyzed by qChIP using the anti-Pol II (8WG16) antibody. Amplicons correspond to sequences upstream of the transcription start site (US −605), transcription start site (TSS −21) and coding regions CR (+1664) and 3′-end (+5411). On the right is reported the ratio of ChIP enrichments relative to TSS amplicon (proximal) and 3′-end (distal). In the bottom panel is reported a similar qChIP analysis with anti-CYCT1 antibody. The ACHR promoter amplicon was used as negative control in all experiments. The values reported were calculated as fold percentage of amount of immunoprecipitated DNA relative to that present in total input chromatin. Error bars indicate the standard deviation from the mean (n = 3).

Previous works have shown that UV induces alteration of Pol II distribution along chromatin of repressed genes. A recent report demonstrated that UV triggers a DDR that inhibits Pol II elongation in vivo [25]. Inhibition of Pol II elongation is fully compatible with the P-TEFb dissociation induced by UV. Since caffeine prevents P-TEFb dissociation we sought to determine the Pol II distribution following UV irradiation in the presence or absence of caffeine. We performed quantitative chromatin immunoprecipitation (qChIP) to test for the occupancy of total Pol II at the single chromatin locus of the pTet-globin-Luc-CFP-24MS2 in 5BCP9F cells. qChIP experiments were carried out using chromatin prepared from untreated, UV treated cells in the presence or absence of caffeine, and the relative occupancy of Pol II was determined by qChIP analysis on upstream (US), transcription start site (TSS, proximal), coding region (CD) and 3′-end region (distal) (Fig. 4). To assess the effects of UV +/− caffeine on Pol II elongation, we used a recent described approach looking at total Pol II distribution and in agreement with a recent report [25] we found that UV treatment causes a decrease in the amounts of Pol II at the pTet-globin-Luc-CFP-24MS2 region, and caffeine partially attenuates this effect. Moreover, as discussed by Munoz et al, [25] if we normalize the −UV, +UV and +UV/caffeine ChIP enrichments to 100% at the TSS (proximal), the Pol II distribution on pTet-globin-Luc-CFP-24MS2 locus in UV-treated cells shows an accumulation toward the TSS proximal region, consistent with an elongation defect. In the presence of caffeine recruitment of Pol II is partially maintained, and most importantly the ratio proximal/distal Pol II recruitment is similar to the untreated samples, suggesting that caffeine prevents, at least in part, inhibition of transcription elongation. In parallel, we performed qChIP to test the chromatin occupancy of P-TEFb, using an anti-CYCT1 antibody, and found that CYCT1 recruitment at the TSS, CR and 3′-end regions decreased (3–4 fold) after UV. Caffeine partially maintained the relative amounts of CYCT1 on gene chromatin following UV.

## Discussion

To sustain UV-induced DNA damage mammalian cells impinge a global reprogramming of gene transcription. Unlike transcription activation the molecular basis of transcriptional repression after DNA damage have not been established fully. UV treatment triggers a rapid disruption of the LC P-TEFb. Concurrently, Rpb1-CTD becomes hyper-phosphorylated and transcription is largely repressed. Our findings showed that caffeine pretreatment of UV-irradiated cells, prevents P-TEFb LC dissociation. Remarkably, caffeine treatment prevents UV-induced Rpb1-CTD hyperphosphorylation and transcription inhibition.

Release of P-TEFb LC complex is a common feature of the DDR induced by a variety of agents: P-TEFb dissociation has been found after UV, drug-induced DNA damage such as ActD and Camptothecin, H_2_O_2_ and albeit transiently, after treatment with the differentiation agent HMBA [13], [18], [21], [24], [26], [27]. The protective effect of caffeine was seen only after UV-induced DNA damage and not after Camptothecin treatment (data not shown), suggesting the presence of different pathways that control transcription in response to different genotoxic insults.

The DDR activated PIKK-kinases, such as ATM, ATR and DNA-PK do not play a significant role in the protective effect by caffeine, because pharmacological inhibition of ATM (KU55933 inhibitor) or DNA-PK (NU7026 inhibitor) as well as ATR mRNA silencing have no effect of P-TEFb dissociation neither rescue transcription. Moreover, we found that the caffeine rescues transcription in UV irradiate cells independently from DNA damage.

Caffeine has a broad inhibitory function of cellular kinases and phosphatases and it also regulates Ca^2^-signaling pathway involved in cell cycle progression and development [23]. It has been shown that treatment with UV or HMBA implicates the calcium-dependent protein phosphatases 2B and 1a, in a process causing the release of P-TEFb from the 7SK ribonucleoprotein complex [24]. It is attractive to hypothesize that caffeine may interfere with UV-induced Ca^2^-dependent activation of calcineurin. Accordingly, we found that the calcineurin inhibitors FK506 and cyclosporine A, both able to prevent P-TEFb LC dissociation [24], reduce transcription inhibition in UV-damaged cells.

As we mentioned above, pretreatment with caffeine did not prevent Campthotecin from disrupting the P-TEFb LC. Thus, a different DDR pathway is involved in this process. Accordingly, several signal transduction pathways appear to control P-TEFb equilibrium. It has been reported that HMBA activates the PI3K/Akt pathway through phosphorylation of HEXIM1 and dissociation of P-TEFb LC [21]. In cardiomyocytes, blockage of Jak/STAT signaling by AG490 prevents release of CLP1 (the mouse HEXIM1 homolog) from P-TEFb [20]. The phosphatase PPM1A and the related PPM1B regulates phosphorylation of CDK9 Thr-186 [28], required for the association and stabilization of the P-TEFb LC complex [29]. Partitioning of active and inactive P-TEFb was also shown by acetylation of cyclinT1 [30]. Finally, it has been suggested that upon transcription arrest 7SK RNA may shuttle between the P-TEFb LC to hnRNPs with a concomitant dissociation of P-TEFb LC [31], [32]. Nevertheless, P-TEFb dissociation is a common response to perturbation of cellular homeostasis by genotoxic stress and which pathway operates is likely dependent on the type of DNA damage inflicted.

It is important to note that caffeine is not fully sufficient to protect transcription at levels comparable to that in untreated cells, suggesting that additional transcriptional regulatory mechanisms are involved in DNA damage transcription arrest. Recent works have indicated that different changes in histone modifications are rapidly induced in response to DNA damage. H3-T11 phosphorylation and H3K9 and K56 acetylation are reduced in response to DNA damage [22], [33]. Moreover, it has been recently reported that UV affects cotranscriptional processing as well as induction of alternative splicing through inhibition of transcription elongation [23]. Interestingly, in a recent report it has been shown that disruption of P-TEFb LC promotes alternative splicing via the transcriptional active P-TEFb [34].

The relative contribution of P-TEFb, histone modifications and mRNA processing following UV-induced DNA damage arrest will be an important issue to decipher the molecular mechanisms underlying the reprogramming of gene expression following DNA damage.

## Materials and Methods

### Cell cultures and drug treatments

HeLa cells were grown in high glucose DMEM with 10% FBS [18]. U2OS/pTet-globin-Luc-CFP-24MS2 cells (internal reference 5BCP9F) were grown in low glucose DMEM with 10% FBS. The 5BCP9F cells were derived from the U2OS Tet-on cells (Clontech) that were co-transfected with pBABE-puro carrying the puromycin resistance gene and pTet-globin-Luc-CFP-24MS2 by standard calcium phosphate procedure. Individual colonies were selected in culture medium containing puromycin (10µg/ml), screened for Luciferase expression after doxycycline induction and subcloned. The pTet-globin-Luc-CFP-24MS2 vector was generated by inserting the PCR amplified Luc gene at the BstXI site of the original pTet-globin-CFP-18MS2-2 construct, harboring 24 MS2 repeats [35]. For UV treatment, exponentially growing cells were irradiated with 254-nm UV light at 40J/m^2^. Pre-treatments with the following inhibitors were added to cells 2 hours before UV treatment, caffeine (2mM), LY294002 (10µM), AG490 (100µM), KU55933 (20µM), wortmannin (50µM) and NU7026 (20µM). In co-treatments experiments cells were first incubated with caffeine, then DRB (50µM) was added to the medium. In luciferase assays 5BCP9F cells were irradiated with UV light and subsequently doxycycline was added at the final concentration of 2µg/ml. Luciferase assays were performed after 4 hours and Luciferase activities were normalized to the cellular protein contents.

### Separation of large and free forms of P-TEFb

HeLa cells were treated with the indicated compounds and glycerol gradient fractionation of cell lysates were carried out as described. Fractions were analyzed by immunoblotting with anti-CDK9. Differential salt extraction of large and free forms of P-TEFb was performed as recently described [18], [19]. Briefly, cytosolic extracts were prepared by resuspending the cells in Buffer A (10 mM KCl, 10 mM MgCl2, 10 mM HEPES, 1 mM EDTA, 1 mM DTT, 0.1% PMSF and protease inhibitor cocktail, Roche) with 0.5% NP-40 for 10 minutes on ice. The nuclei were spun down and the supernatant was saved as the cytosolic extract (CE). The nuclei were washed and resuspended in Buffer B (450 mM NaCl, 1.5 mM MgCl2, 20 mM HEPES, 0.5 mM EDTA, 1 mM DTT, 0.1% PMSF), lysates were clarified by centrifugation, and the supernatant was saved as the nuclear extract (NE). Samples were analyzed by immunoblotting. The antibodies used for western blotting were: anti-CYCT1 and anti-CDK9 (both from, Santa Cruz), Glycerol gradients were performed as previously described [25].

### siRNA treatments

siRNA experiments in 5BCP9F cells were carried out using MicroPorator Digital Bio Technology. Indicated siRNA were introduced into each 3×10^6^ dissociated cells in 100 µl volume according to manufacturer's instructions. After 48 hrs of recovery, doxycycline was added and the cells were irradiated with UV as indicated in the text. For siRNA treatments, ON-TARGETplus SMARTpool ATR; (L-003282-00-0005) and ON-TARGETplus Non-targeting pool (D-001810-10-5) were obtained from Dharmacon. 100 nM, final concentration of the pools was used for each transfection. Expression of siRNA target genes was evaluated by qRT-PCR and proteins levels were determined by western blot.

### qRT-PCR and Quantitative Chromatin Immunoprecipitation (qChIP)

cDNA was prepared from total RNA with Quantitect Reverse Transcription Kit (Qiagen) according to manufactory instructions. Each sample was assayed in triplicate, and the qRT-PCR data were normalized to the expression of the housekeeping beta-glucuronidase (GUS). qChIP experiments were performed essentially as described [36]. Antibodies used in these experiments were as follow: anti-CYCT1 (T-18, Santa Cruz) anti-Pol II (8WG16, Covance). For qPCR 3µl out of 150µl immunoprecipitated DNA was used with primers upstream (US -605), transcription start site (TSS, −21, proximal), coding region (CD +1664) and 3′-end region (+5411 distal). The ACHR promoter amplicon was used as negative control in all experiments, primer sequences are available on request. Normal serum and input DNA values were used to subtract/normalize the values from qChIP samples. All qChIP data derived from at least three independent experiments.

### Fluorescence In Situ Hybridization (FISH) and immunofluorescence

Immunofluorescence and FISH were performed as described previously on paraformaldehyde-fixed cells. MS2 oligodeoxynucleotide containing 5-amino-allyl thymidines (Eurogentec) was labeled with Cy3 (GE Healthcare).

## Supporting Information

Figure S1 Panels A and B: HeLa cells were irradiated with UV (40J/m^2^), and at the indicated times (mins) in the absence (A) or presence of caffeine 2hr of pretreament (panel B) after irradiation, cellular proteins were extracted with different buffers as described in the text, and immunoblotting was performed on low cytosolic extracts (CE) and high-salt nuclear extracts (NE). On the left, a graph reports the relative quantification of the immunoblots as a percentage of large P-TEFb complex. Graphs are representative of at least four independent experiments; error bars represent standard deviation from the mean (n = 3). The percent of P-TEFb in large complex (low salt or CE) was calculated as a fraction of total amount of P-TEFb (both in CE and NE). On the right, western blots from a single experiment are shown. Panel C: HeLa cells were treated with caffeine (2mM) for different times (20′ 60 and 120′), and the on the left, a graph reports the relative quantification of the immunoblots as percentage of large P-TEFb complex. Graphs are representative of at least four independent experiments; error bars represent standard deviation from the mean (n = 2). On the right, western blots from a single experiment are shown.
(1.44 MB TIF)
