# Correction: Caffeine Prevents Transcription Inhibition and P-TEFb/7SK Dissociation Following UV-Induced DNA Damage

**DOI:** 10.1371/annotation/753c6a63-2aea-4cb8-b38c-28db972d60df

**Published:** 2013-10-07

**Authors:** Giuliana Napolitano, Stefano Amente, Virginia Castiglia, Barbara Gargano, Vera Ruda, Xavier Darzacq, Olivier Bensaude, Barbara Majello, Luigi Lania

Panel B of Figure 1 in the article is a composite figure in which the first lanes (untreated samples) are derived from different blots. We are supplying the raw blots for this figure as part of this Correction.

Raw Blots: http://plosone.org/corrections/pone.0011245.g001.cn.tif

As can be seen from the provided raw data, the original blots contain additional irrelevant control lanes, which were eliminated to prepare the final Figure 1 panel B, the results and conclusions reported in the article are not affected by this modification. The raw data show that the results presented in Figure 1 panel B are from a single experiment. 

