# Supplementary figures and images for: Caffeine Prevents Transcription Inhibition and P-TEFb/7SK Dissociation Following UV-Induced DNA Damage
Source: PLoS One. 2010 Jun 21;5(6):e11245. doi: 10.1371/journal.pone.0011245 (PMC2888590; doi:10.1371/journal.pone.0011245)

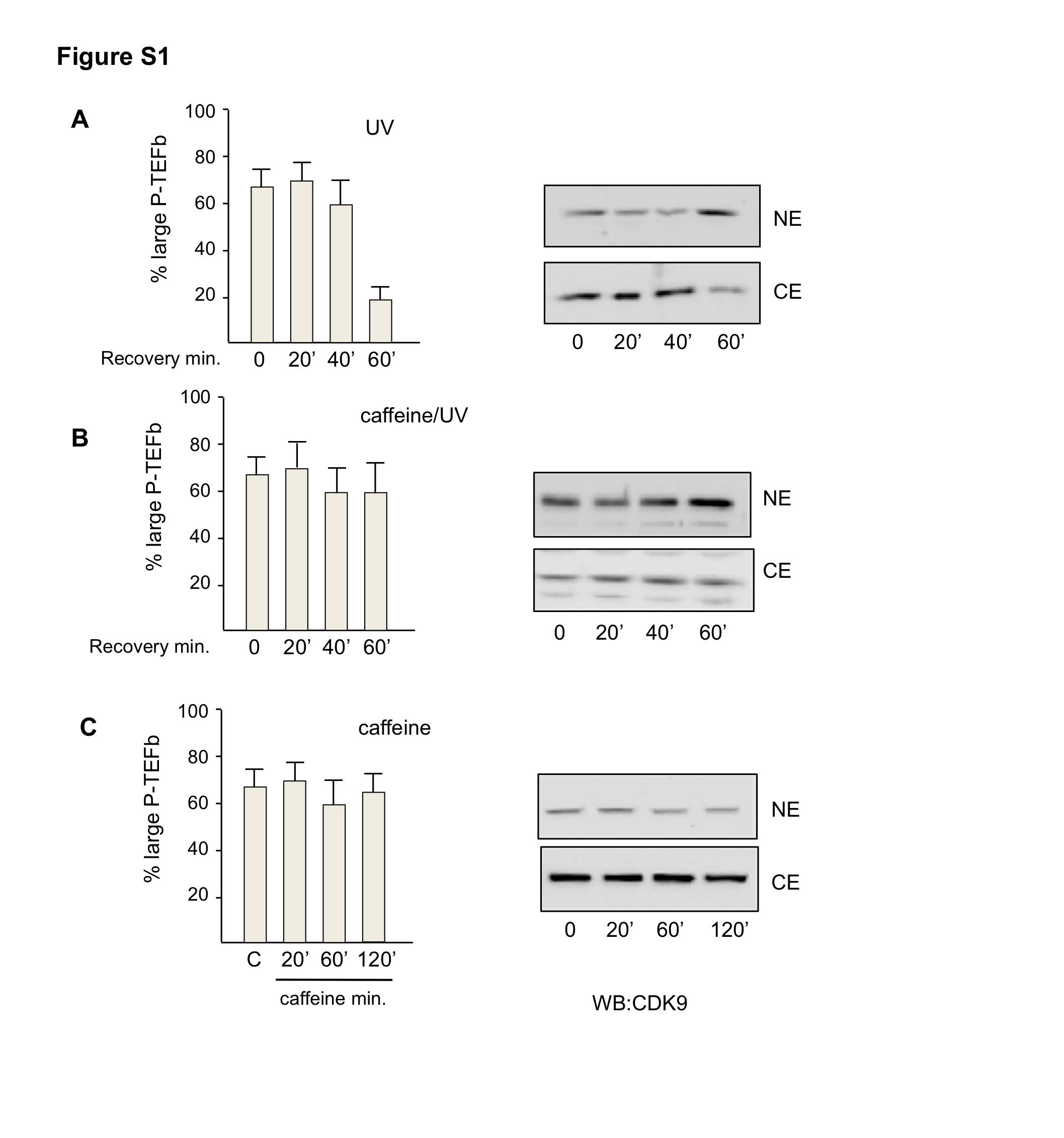

Supplement: Figure S1 — Panels A and B: HeLa cells were irradiated with UV (40J/m2), and at the indicated times (mins) in the absence (A) or presence of caffeine 2hr of pretreament (panel B) after irradiation, cellular proteins were extracted with different buffers as described in the text, and immunoblotting was performed on low cytosolic extracts (CE) and high-salt nuclear extracts (NE). On the left, a graph reports the relative quantification of the immunoblots as a percentage of large P-TEFb complex. Graphs are representative of at least four independent experiments; error bars represent standard deviation from the mean (n = 3). The percent of P-TEFb in large complex (low salt or CE) was calculated as a fraction of total amount of P-TEFb (both in CE and NE). On the right, western blots from a single experiment are shown. Panel C: HeLa cells were treated with caffeine (2mM) for different times (20′ 60 and 120′), and the on the left, a graph reports the relative quantification of the immunoblots as percentage of large P-TEFb complex. Graphs are representative of at least four independent experiments; error bars represent standard deviation from the mean (n = 2). On the right, western blots from a single experiment are shown. (1.44 MB TIF) [file pone.0011245.s001.tif]
